# A Shot That Hits the Tumor: Incidental Finding of Early Colon Cancer in a Gunshot Wound Specimen—The Role of Pathologic Examination

**DOI:** 10.1155/2019/8059346

**Published:** 2019-04-15

**Authors:** Neda Soleimani, Sara Pakbaz

**Affiliations:** ^1^Department of Pathology, Shiraz University of Medical Sciences, Shiraz, Iran; ^2^Department of Pathology, Toronto General Hospital, University of Toronto, Canada

## Abstract

This report presents incidental finding of early colorectal cancer in an adult patient with gunshot injury. The patient was a 41 y/o man, transferred to our center due to gunshot wound to his abdomen and back. A well differentiated adenocarcinoma, stage I, was incidentally identified during pathologic examination on his segmental proctectomy specimen. This singular case highlights the necessity of caring for all removed tissues, indicating how important they are for both clinicians and pathologists.

## 1. Introduction

Colorectal cancer is a main cause of morbidity and mortality throughout the world [1]. It is the third most common cancer worldwide and the fourth most common cause of death [2]. Patient survival is highly dependent upon stage of disease at diagnosis. We would like to present incidental finding of early rectal cancer (stage I) in a 41 y/o man with gunshot wound to his abdomen.

## 2. Case Report

A man aged 41 years was transferred to our center at Shiraz University of Medical Sciences, Iran, sustaining gunshot wound to his back and abdomen. In terms of past medical history, he was a rural man with poor healthcare and traditional drug addict (flexible dosing) with no family history of cancer. There was not any history of weight loss, anorexia, and change in bowel habit. Although he was hemodynamically stable, initial evaluation showed retroperitoneal hematoma (about 500 cc blood) with expansion from zone III to zone I and also S2 vertebral fracture.

At the laparotomy, patchy necrosis of rectum was detected and short segmental resection (5.5 cm in length) was performed. Although, there was not any gross evidence of abnormal finding during operation, as a rule in our department any specimen removed from human body should be sent to pathology examination and the specimen underwent pathologic assessment.

**Figure 1:**
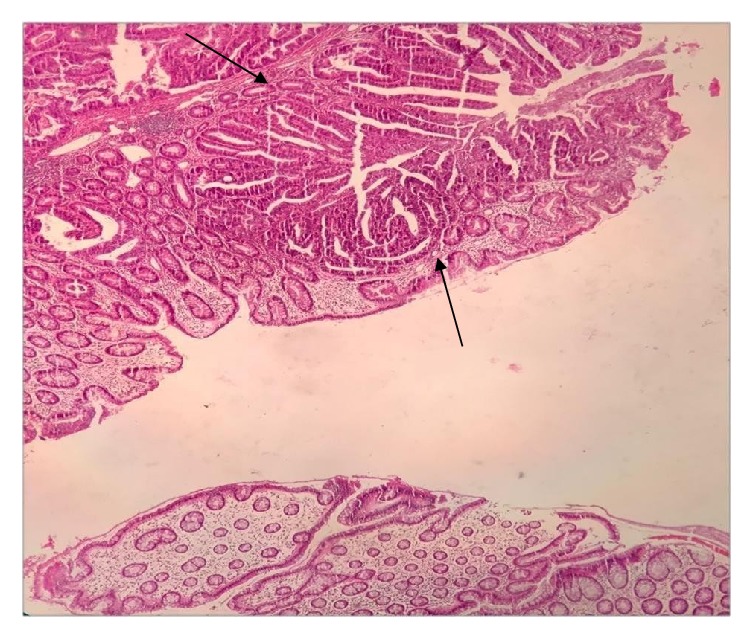
Well differentiated adenocarcinoma at one margin of colon specimen (arrow) (H&E x 10).

**Figure 2:**
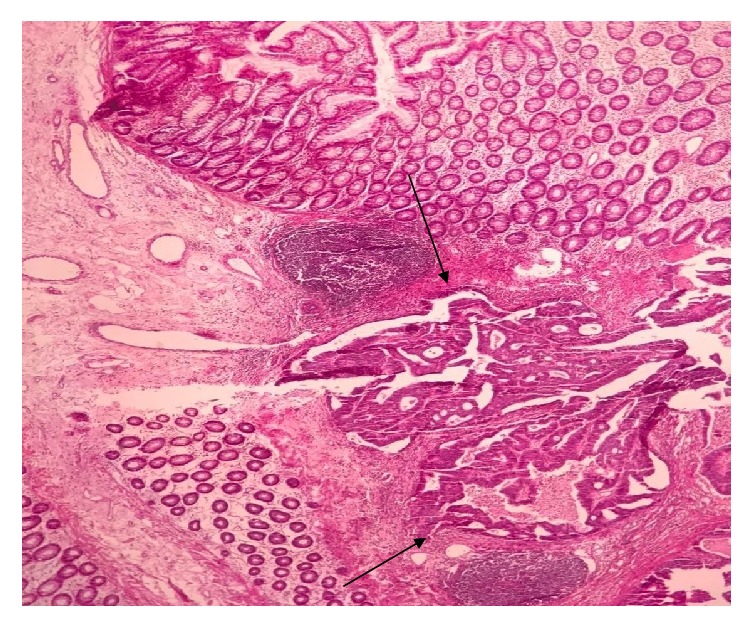
Tumor invades to submucosa (arrow) (H&E x 20).

Grossly there was edema, multifocal necrosis, and a small polypoid firm lesion measuring 1x1x0.5 cm near one margin, histologically showing well differentiated adenocarcinoma, hard to believe (Figure 1). Microscopic tumor extension was limited to submucosa (T1), signed out as stage I (Figure 2).

## 3. Discussion

Surgical pathology is the study of tissues removed from patients, covering physical examination of tissue with naked eye as well as microscopic examination. Any specimen would be potentially considered as malignant, and even very experienced pathologists are often surprised when comparing the macroscopic diagnosis with the histopathologic slides. Therefore careful orientation, gross examination, and proper sectioning of surgical specimens are mandatory. It is not hard to diagnose malignancy through microscopic examination if the sections are taken from proper sites. Although histologic examination of the vast majority of trauma specimens shows signs of acute inflammation, sometimes it is done to check the viability of resected margins as done in our case [3]. The surgeons should always keep in mind that it is important to follow the pathologic reports in all cases, not just because there might be malignancy (like ours), but sometimes other pathologies that may require further postoperative managements are found. Examples include incidental finding of Rosai-Dorfman disease in tonsillectomy specimen of a 4-year-old girl, primary fallopian tube carcinoma during bilateral salpingooophorectomy for removal of ovarian cyst, and gall bladder adenocarcinoma during pathological evaluation of cholecystectomy specimens [4–6]. Once we found unexpected malignancy—in fact any unexpected pathology is a critical value—we called a surgeon at the same time to do better evaluation [7]. Fortunately, in the lesion that we found, microscopic tumor extension was just limited to submucosa, without any lymph node involvement or metastasis, so it was signed out as stage I.
